# Pseudosarcomatous Fibromyxoid Tumor of the Prostate: A Rare Benign Lesion Mimicking Malignancy

**DOI:** 10.7759/cureus.56594

**Published:** 2024-03-20

**Authors:** Kavita Somani, Pretty Singh

**Affiliations:** 1 Department of Pathology and Laboratory Medicine, Apollomedics Super Speciality Hospital, Lucknow, IND

**Keywords:** fibromyxoid, psuedosarcoma, genitourinary, prostate, psft

## Abstract

Psuedosarcomatous fibromyxoid tumor (PSFT) is an uncommon, non-malignant yet locally aggressive pseudotumor found in the genitourinary system. Despite being a benign spindle cell tumor without any documented cases of metastasis, its local aggressiveness can pose a life-threatening risk. The lack of specific clinical symptoms and the infiltrative characteristics of the lesion may lead to misdiagnosis as sarcomatoid carcinoma or sarcoma. Therefore, it is crucial to distinguish PSFT histologically and through immunohistochemistry from other spindle cell tumors to avoid unnecessary investigations and treatments.

This case emphasizes the difficulties in diagnosing this uncommon benign tumor because of its infrequent occurrence, limited literature, vague symptoms, and similarities in imaging results with inflammatory or infectious conditions, as well as sarcomatous neoplasms. Precise diagnosis plays a vital role in preventing unnecessary or insufficient treatment.

## Introduction

Pseudosarcomatous fibromyxoid tumor (PSFT) of the prostate is an exceedingly rare benign entity that can mimic malignant lesions both clinically and histopathologically.

In 1980, Roth initially reported on the presence of pseudosarcoma in the genitourinary tract [1]. This reactive pseudosarcomatous response is an infrequent benign lesion found in the urinary tract. It is referred to by various names in the literature, such as nodular fasciitis, pseudosarcomatous myofibroblastic tumor, fibromyxoid pseudotumor, and others [2]. The most commonly referred names are PSFT or postoperative spindle cell nodule (PSCN). The two lesions can be separated only by a history of surgical instrumentation or previous surgery of the urinary tract in the latter.

Here, we present the case of a 42-year-old man who presented to our tertiary care center with a complaint of lower abdominal pain along with urinary tract symptoms and was found to have a PSFT infiltrating the prostate and both seminal vesicles.

We wish to present this case given its rare occurrence, lack of relevant literature, and benign yet aggressive nature of the neoplasm.

## Case presentation

**Figure 1:**
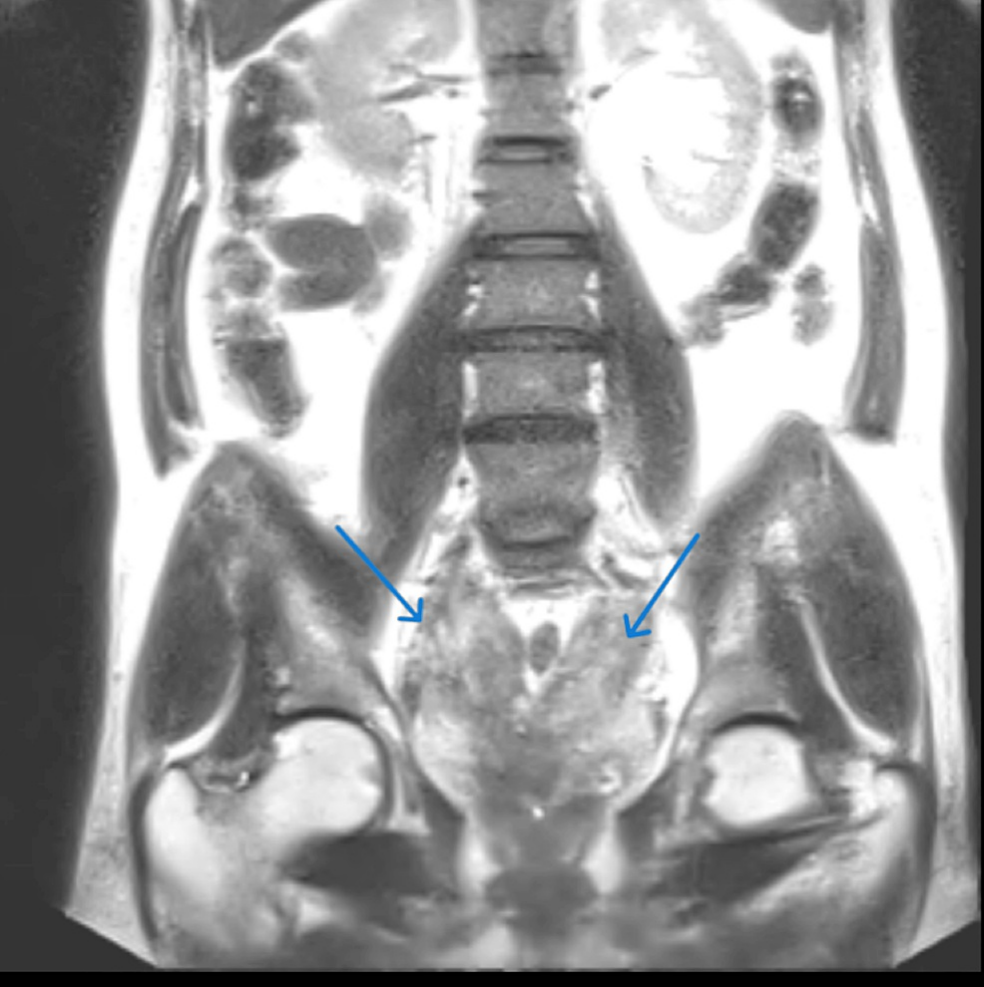
MRI of the pelvis showing a mass involving bilateral seminal vesicles and abutting the posterior wall of the urinary bladder.

A 42-year-old male patient presented at the urology department with complaints of lower urinary tract symptoms and pelvic pain. Magnetic resonance imaging (MRI) revealed a mass-like lesion involving bilateral seminal vesicles, abutting the posterior wall of the urinary bladder and bilateral pelvic fascia laterally, as well as encasing both distal ureters, resulting in features of obstructive uropathy and raising suspicion of chronic inflammatory or infective etiology (Figure 1).

The patient had a serum creatinine level of 4 mg/dL and moderate-grade bilateral hydronephrosis. Given the obstruction caused by the periureteric mass, which was compressing the ureters, bilateral stents were placed and the patient was started on conservative management to improve the renal function.

Whole-body positron emission tomography-computed tomography (PET-CT) revealed a hypermetabolic enlarged prostate along with a hypermetabolic ill-defined lesion, involving the bilateral ureters and seminal vesicles, suggestive of an inflammatory etiology.

The serum prostate-specific antigen was 2 ng/mL. Considering a large mass infiltrating the prostate, a transrectal ultrasound-guided (TRUS) biopsy of the prostate was performed. Histopathological examination showed a spindle cell lesion with smooth muscle actin positivity. Hence, a diagnosis of spindle cell neoplasm was made.

This finding raised concerns due to the large size of the mass along with the patient’s clinical symptoms. Consequently, laparoscopic radical prostatectomy with bilateral seminal vesicles, along with excision of the pelvic mass and both lower ureteric margins, was performed for comprehensive evaluation and management.

**Figure 2:**
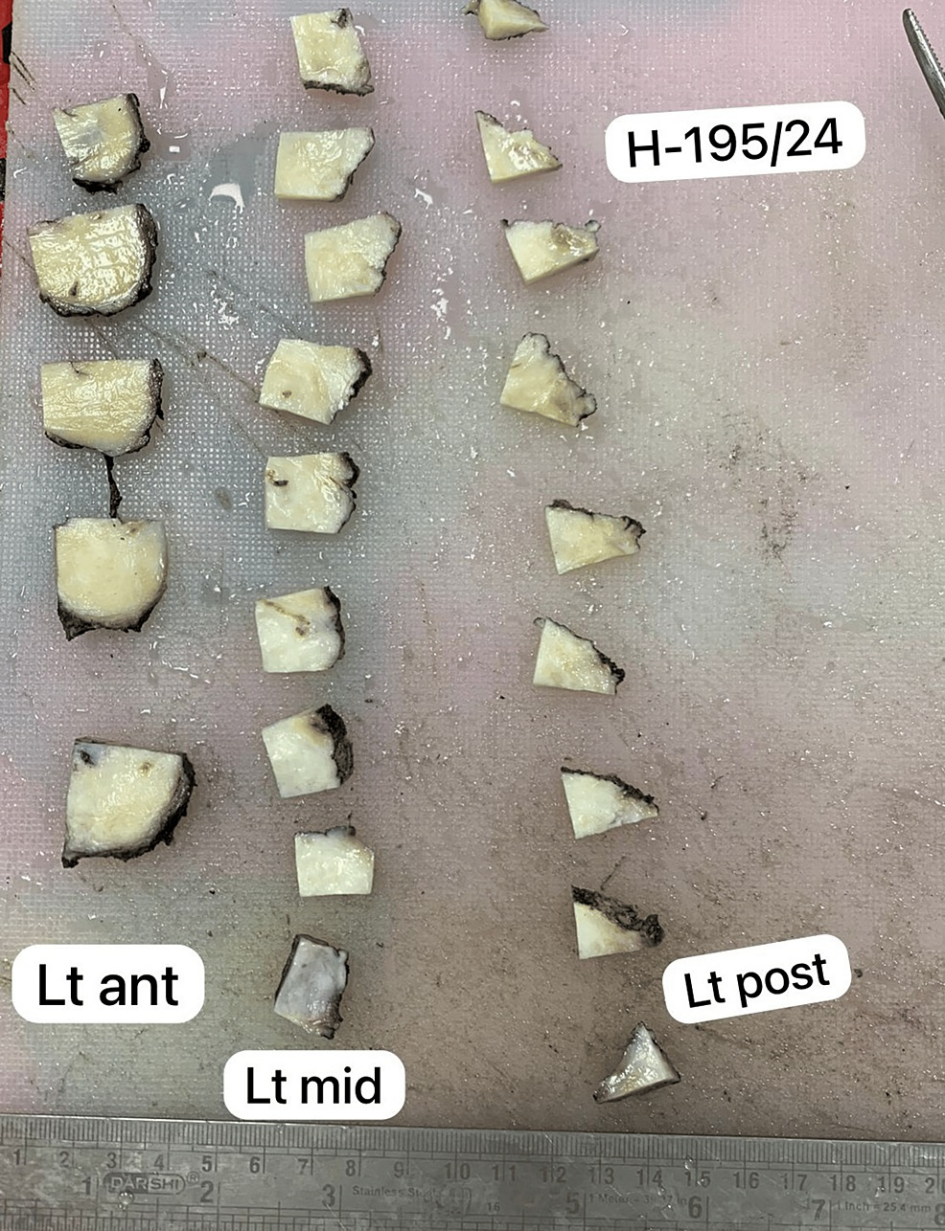
Serial sections of the prostate showing myxoid yellowish-white areas.

**Figure 3:**
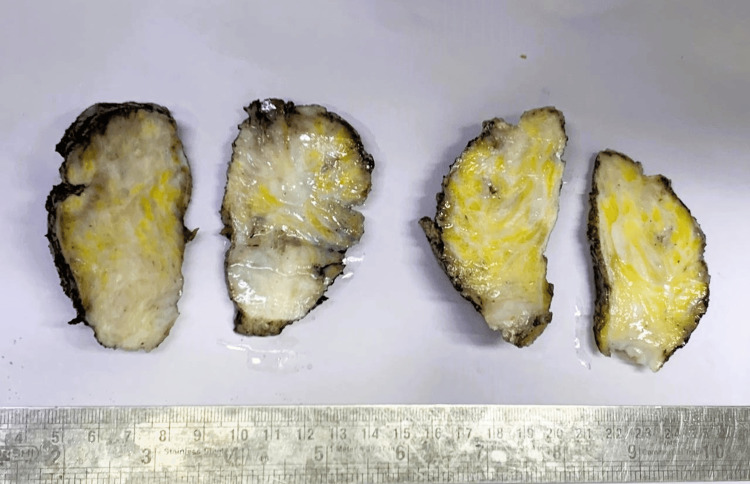
Bilateral enlarged seminal vesicles showing yellowish-white myxoid areas.

On gross examination, radical prostatectomy with bilateral seminal vesicles measured 6.0 × 5.0 × 4.1 cm. Cut sections showed diffuse gray-white to gray-yellow myxoid areas measuring 4.5 × 3.0 × 3.0 cm involving the posterior part of the prostate as well as both seminal vesicles (Figures 2, 3).

**Figure 4:**
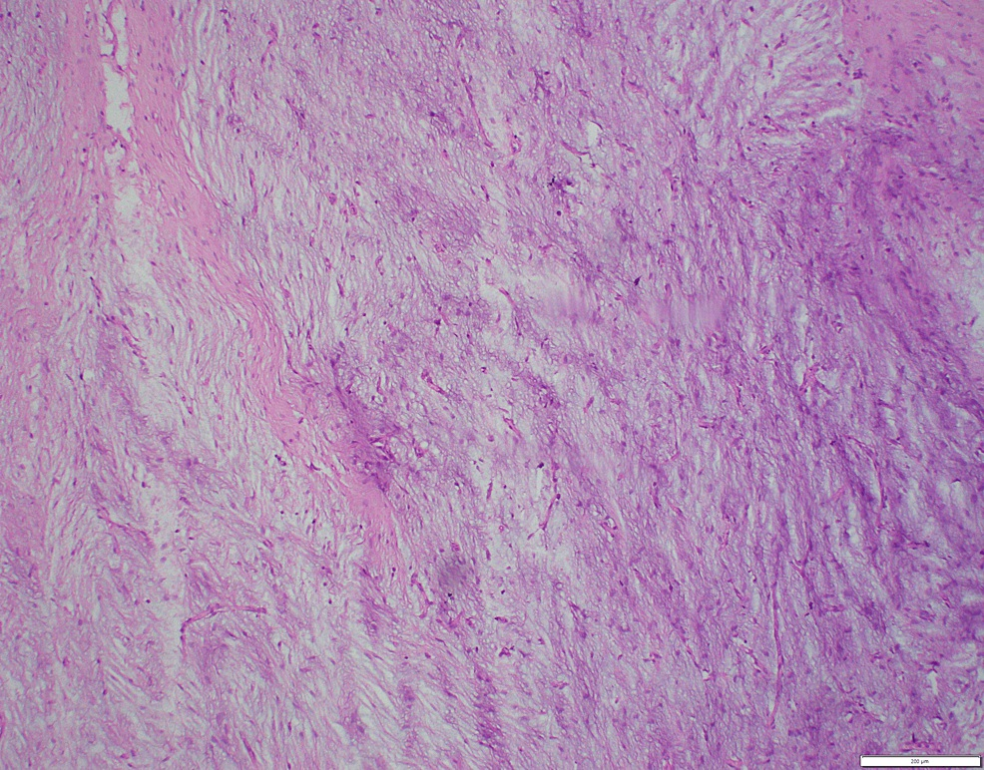
Hematoxylin and eosin section from the prostate (100×): myxoid areas with stellate cells.

Histological examination of the excised tissue demonstrated a well-demarcated, unencapsulated fibromyxoid tumor involving the prostate and both seminal vesicles. The tumor exhibited myxoid stroma with interspersed stellate to spindle-shaped cells along with thin-walled blood vessels. No cytological atypia, mitotic figures, or necrosis was observed (Figure 4).

**Figure 5:**
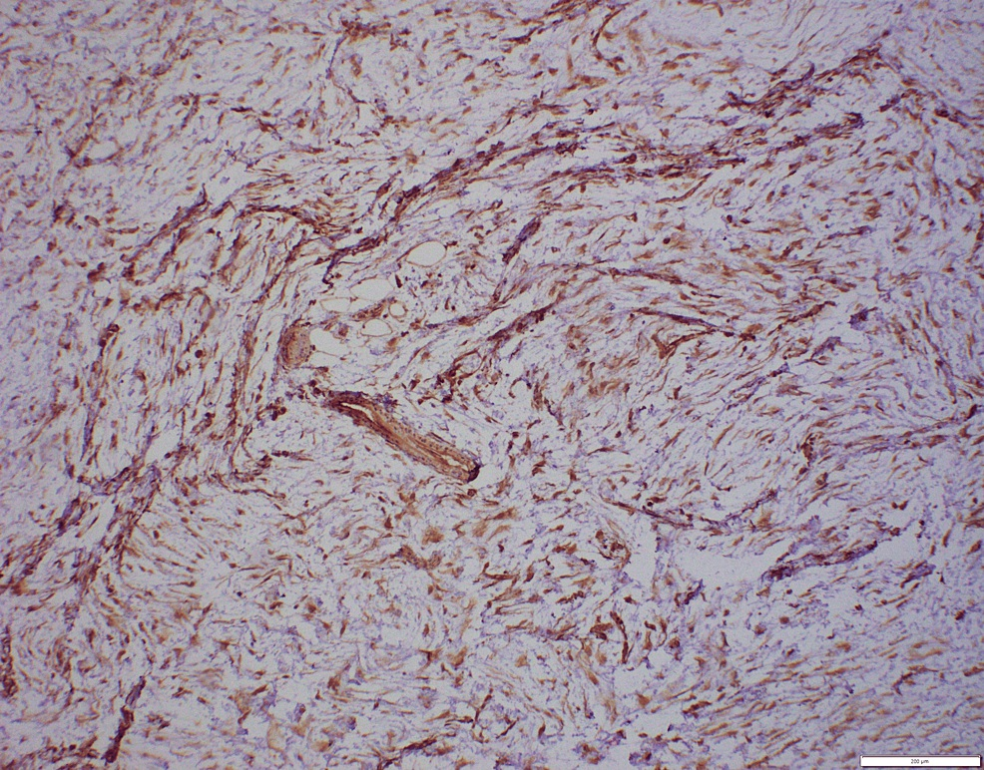
Immunohistochemistry. Vimentin (100×): positive in tumor cells.

**Figure 6:**
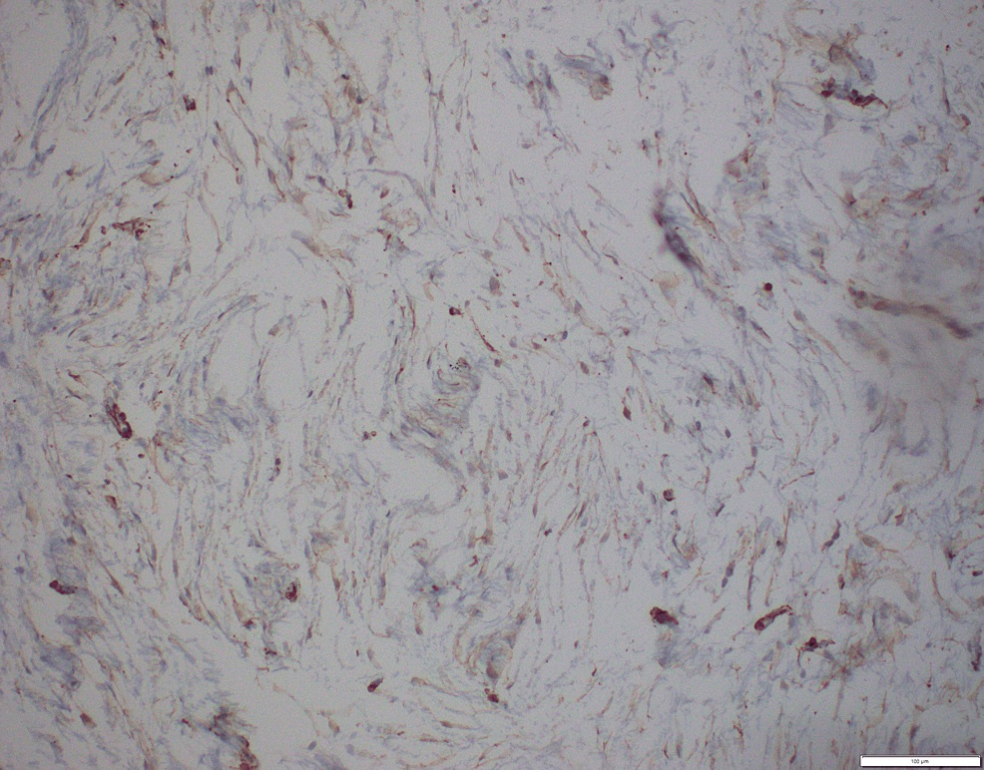
Immunohistochemistry. Smooth muscle actin (100×): tumor cells showing cytoplasmic positivity.

**Figure 7:**
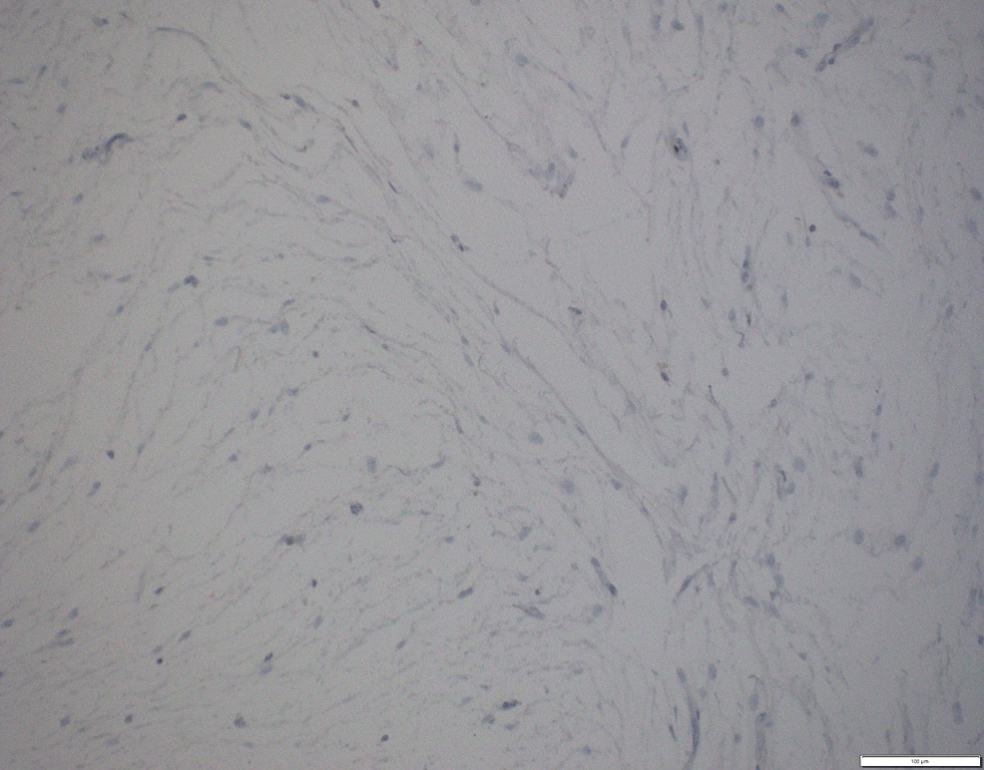
Immunohistochemistry. Ki67 (200×): low proliferation index.

An immunohistochemistry assessment was subsequently performed, which revealed positive staining for vimentin and SMA, indicating smooth muscle differentiation, and a low Ki-76 proliferation index (1-2%). Negative staining for S-100 protein, ALK1, desmin, myogenin, and cytokeratin ruled out nerve sheath tumor, inflammatory myofibroblastic tumor, leiomyosarcoma, and rhabdomyosarcoma, respectively. Thus, the final diagnosis was PSFT of the seminal vesicle/prostate (Figures 5-7).

## Discussion

Pseudosarcomas occurring in the genitourinary tract are uncommon abnormalities, and their underlying causes remain poorly understood. The diagnosis of these tumors is established through histological examination, which reveals the presence of stellate and spindle cells proliferating within a myxoid stromal background, without any notable increase in mitosis [3].

In 1980, Roth initially documented a spindle cell lesion situated in the urinary bladder, referring to it as a “reactive pseudosarcomatous response.” Four years later, in 1984, Hafiz et al. reported a comparable lesion in the prostate [4]. Several case studies have been documented in the literature regarding PSFT of the bladder and prostate whereas a limited number of cases involving the ureter, vagina, and urethra have been reported [5-7]. Ro et al. first used the term “pseudosarcomatous fibromyxoid tumor” for these types of lesions of the prostate and urinary bladder [8].

Due to the uncommon occurrence of the neoplasm, there is a scarcity of extensive literature, leading to inconsistent nomenclature and categorization. The commonly employed terms to refer to these growths are PSFT and PSCN. These lesions share identical histological features, demonstrate a comparable prognosis, and can be successfully managed through surgical removal alone.

The cause of PSFTs remains unclear. However, in many documented cases, there have been common factors such as smoking, previous urogenital tract surgery, or instrumentation. In our case, smoking was the only identified probable risk factor for the development of the pseudosarcomatous tumor.

One notable characteristic of PSFTs is their remarkably fast growth rate. When considering the differential diagnosis, it is important to consider sarcomatoid urothelial carcinoma and sarcomas such as leiomyosarcoma and rhabdomyosarcoma due to mesenchymal proliferation [9]. A study revealed that the main characteristics distinguishing sarcomas from pseudosarcomatous tumors were histologic features such as nuclear atypia, mitotic figures, and the presence of necrosis or myxoid degeneration. Differentiating between pseudosarcomas and sarcomas is crucial, as the latter necessitates a more extensive and costly evaluation, along with advanced and aggressive treatment [10]. In contrast to sarcomas, metastasis has not been documented with PSFT despite their potential for aggressive local invasion and rapid growth.

Local excision is the treatment of choice for both PSCN and PSFT, either by prostatectomy, transurethral resection (TUR), or radical or partial cystectomy, depending upon the organs involved. However, recurrence has been reported in cases treated with TUR, for which various theories have been suggested, inferring that it could be due to the persistence of a lack of negative margins rather than an actual recurrence. Fatalities resulting from this illness are infrequent and typically attributed to obstructive uropathy with urosepsis [11]. Jensen et al. presented a case study involving a PSFT of the prostate. The patient in their study underwent an additional transurethral resection of the prostate due to worsening lower urinary tract symptoms [12]. Similarly, Harik et al. examined 42 cases of PSFTs of the bladder. The findings revealed that while some patients experienced recurrences, none developed metastases [13].

In our case, MRI and PET findings initially suggested chronic inflammatory or infective involvement due to bilateral hydronephrosis and bulky heterogeneous seminal vesicles. However, the TRUS biopsy revealed no tumor but a benign spindle cell lesion. The decision for radical prostatectomy, in conjunction with the excision of the pelvic mass and both ureteric margins, was prompted by the clinical presentation and suspicion of an underlying aggressive process, which was proven to be PSFT on histopathological examination. Our patient remained asymptomatic even after two months of surgery, without requiring any additional treatment.

## Conclusions

PSFT of the prostate is an uncommon non-malignant growth that can imitate malignant tumors both in terms of clinical presentation and histopathological features. Accurately diagnosing this condition can be challenging due to similar imaging findings seen in infectious or inflammatory conditions. Therefore, it is crucial to consider this specific tumor during the diagnostic process to avoid unnecessary aggressive treatments. Immunohistochemistry plays a significant role in confirming the diagnosis and distinguishing this benign lesion from malignant tumors. Further research and case reports are necessary to enhance our understanding of the tumor’s clinical behavior and determine the optimal diagnostic approach.

Due to the rare occurrence of pseudosarcomatous lesions in the urinary tract, there have only been a few reports regarding epidemiological data, clinical presentation, and histological characteristics of this lesion. In conclusion, PSFTs of the prostate are rare lesions, and urologists and pathologists need to distinguish this benign process from a malignant lesion to avoid unnecessary radical procedures.
